# The Immunoregulation of Th17 in Host against Intracellular Bacterial Infection

**DOI:** 10.1155/2018/6587296

**Published:** 2018-03-19

**Authors:** Yonghong Li, Chaojun Wei, Hui Xu, Jing Jia, Zhenhong Wei, Rui Guo, Yanjuan Jia, Yu Wu, Yuanting Li, Xiaoming Qi, Zhenhao Li, Xiaoling Gao

**Affiliations:** The Institute of Clinical Research and Translational Medicine, Gansu Provincial Hospital, Lanzhou 730000, China

## Abstract

T helper 17 cells (Th17) constitute a distinct subset of helper T cells with a unique transcriptional profile (STAT3, ROR*γ*, and ROR*α*), cytokine production pattern (IL17 family), and requirement of specific cytokines for their differentiation (TGF-*β*, IL6, IL21, and IL23). Recent studies involving experimental animals and humans have shown that Th17/IL17 plays a crucial role in host defense against a variety of pathogens, including bacteria and viruses. The underlying mechanisms by which Th17 performs include dendritic cell (DC) regulation, neutrophil recruitment, Th1 modulation, and T regulatory cell (Treg) balance. In recent years, researchers have generated an accumulating wealth of evidence on the role of Th17/IL17 in protective immunity to intracellular bacterial pathogens, such as *Mycobacterium tuberculosis* and *Chlamydia trachomatis*, which are one of the most important pathogens that inflict significant socioeconomic burden across the globe. In this article, we reviewed the current literature on the functions and mechanisms by which Th17/IL17 responds to intracellular bacterial infections. A better understanding of Th17/IL17 immunity to pathogens would be crucial for developing effective prophylactics and therapeutics.

## 1. Background

The current field of medicine was marked not only by an increase in the endemism of socially significant infectious diseases but also by an action taken to fight against them globally. Despite active antiepidemic actions, mass vaccination campaigns, and facilities of etiotropic therapy, infectious diseases still challenge the whole world. Understanding the essence of infectious pathogenic factors of diseases at the cellular and molecular level will allow us to form a holistic view of the anti-infectious immune reactivity.

It is estimated that over one billion patients are infected with intracellular bacteria that infect and replicate inside host cells, which may be facultative or obligatory. Facultative intracellular bacteria include *Listeria monocytogenes*, *Salmonella* spp., *Francisella* spp., and *Legionella* spp., while obligate intracellular bacteria, such as *Chlamydia* spp., generally require a host cell for survival and replication. It is believed that the acquired resistance against intracellular bacteria depends on CD8^+^ T cells. However, current scientific and technological development has deepened our understanding of host immunity against the intracellular bacteria.

After bacterial invasion, innate immunity provides the initial acute inflammatory response to microorganisms to prevent, control, and eliminate the infection and further modulate and stimulate adaptive immune responses. The acute inflammatory response is generally self-limiting and results in tissue repair, while the persistent inflammatory stimuli or dysregulation of immune mechanism results in chronic inflammation, recognized as a different kind of cytokine/chemokine secretion to activate and attract immune cells into invasive sites. Antigen-presenting cells, like DC and macrophage, are central players in the immune response. After activation, they migrate to lymphoid organs to present antigen to naive T cells and initiate Th cell differentiation [1, 2]. Classically, Th1 response (IL12/IFN-*γ*) is crucial for host defense against intracellular infection by activating cellular immunity to kill bacterial and infected cells [3, 4]. Current advances in the understanding of intracellular bacterial infection indicate that immune response is more complex than the Th1/Th2 paradigm [5]; the recent recognition of Th17 cells has provided new insights into the mechanisms that are important in antimicrobial host defense [6–8].

Th17 cells potentially induce tissue damage and have been associated with many autoimmune diseases and extracellular pathogen infection [9–11]. But several lines of evidence suggested that Th17 cells are also required for host defense against intracellular bacterial infection, such as *L. monocytogenes* [12], *M. tuberculosis* [13–17], *Chlamydia* [18], *Salmonella* [19, 20], *Mycoplasma pneumoniae* [21], and *Leishmania donovani* [22]. Indeed, mice deficiency in both IL23 and IL12 are more susceptible to *M. tuberculosis* and *Toxoplasma gondii* infection compared to the IL12 knockout mice.

Th17 differentiation depends on the steroid receptor-type nuclear receptor (ROR*γ*t), which is induced by the IL6 and IL23 through activation of signal transducer and activator of transcription (STAT3) [23]. IL21 can promote Th17 cell differentiation, survival, and expansion. In addition, IL23, originally regarded as the key Th17 inducer, is only required for its expansion and maintenance [24]. In addition to STAT3 and ROR*γ*, many other transcript factors play a critical role in Th17 differentiation, including basic leucine zipper ATF-like transcript factor (BATF), IRF4, fos-related antigen 2 (FOSL2), and ROR*α* [25–29]. Th17 cells are reciprocally related to FoxP3^+^ Tregs. The presence of a high dose of TGF-*β* activates the transcription factor FoxP3 in naïve T cells and thereby promotes Treg, whereas the presence of IL6 suppresses FoxP3. It, combined with TGF-*β*, induces ROR*γ*t and leads to Th17 differentiation. Thus, the balance between Th17 cells and FoxP3^+^ Tregs is mediated by the antagonistic interaction of the transcription factors FoxP3 and ROR*γ*t [30]. A recent study has added further complexity to the Th differentiation. It indicated that the CD4^+^ T cells could end with continuous cell fates, rather than a limited number of distinct phenotypes, when exposed to numerous combinations of cytokines [31].

Th17 cells are named from IL17 secretion by the Th17. The activation of Th17 cells results in a large amount of inflammatory cytokine production, such as IL17A, IL17F, IL21, IL22, and CCL20 [11, 32]. IL17 acts as the most critical biological effector of Th17. IL17 increases iNOS production and induces the expression of granulocyte macrophage colony-stimulating factor, IL1*β*, IL6, IL8, TNF, and several chemokines. IL17A and IL17F have a high similarity in sequence and share many biologic properties. Along with IL17, Th17 cells also produce IL22, IL26, and GM-CSF. IL22 and IL26 are members of the IL10 family with significantly different biological activities despite their similar structure. IL22 and IL26 can support tissue reactions of innate immunity and simulate the production of IFN*γ* and secretion of antimicrobial peptides, including acute phase proteins, such as serum amyloid A, A1-antichymotrypsin, and haptoglobin. Furthermore, some study shows that IL22 is a potent stimulator of Th2 responses and can directly upregulate the expression of epithelial-derived type 2 cytokines, such as IL-33, TSLP, and GRP, thus promoting a strong Th2-biased systemic immune response [33]. In contrast to IL17 and TNF*α*, rather work on immunocompetent cells, IL22 acts essentially on epithelial cells and hepatocytes. It is known that IL26 is involved in local mucosal immunity. Monocytes stimulated with IL26 promote Th17 cell development. GM-CSF is produced by Th17 cells to activate macrophages and play a role in immunity against anti-intracellular pathogen [34].

The biological activities of IL17 depend on their binding to its multimeric receptors [35]. The IL17 receptor family contains at least five members: IL17RA, IL17RB, IL17RC, IL17RD, and IL17RE [36]. To date, IL17A and IL17F have been shown to form either homodimers or heterodimers that can bind to the IL17RA and IL17RC receptor complex to activate downstream signaling cascades, whereas IL17E is believed to signal though the IL17RA and IL17RB receptor complex to activate its downstream pathways [37]. At present, the IL17RA and IL17RE heterodimer is considered the functional receptor for IL17C. And IL17RB has been suggested to be a receptor for IL17B [38]. Moreover, IL17RD was recently found to be a positive component in IL17 signaling and a negative suppressor for TLR signaling [39, 40]. The differences in the cytokine-receptor combination largely shape the functional diversity of this family of cytokines at distinct barrier surfaces. The necessary cytokines involved in the Th17 differentiation in mice and human are not identical. TGF-*β* and IL6 are required for mouse Th17 development, whereas human naive T cells differentiate into Th17 cells in the presence of IL1*β*, IL23, and possibly TGF-*β* [41]. The cooperation of all inflammatory factors potentiates tissue inflammation, and the consequences of host immune responses depend on the pathogens.

The IL17 production is multicellular in origin. *γδ* T cells have been found to contribute to the early production of IL17 in a murine model of some intracellular bacterial infection, like *M. tuberculosis* [42], *M. bovis*, *BCG* [15], *Listeria monocytogenes* [43, 44], *S. enterica* [45], and *S. typhimurium* [46]. Even some studies suggested that dominant cellular source for IL17 production is *γδ* T cells, rather than Th17 [42, 44]. Furthermore, a *αβ* TCR^+^ CD4^−^CD8^−^ double negative T cell population which produced IL17 has been found in *L. monocytogenes* [43] and *F. tularensis* LVS infection [47]. Invariant natural killer T (iNKT) cells were able to produce IL17 after stimulated with lipopolysaccharide [48]. iNKT cells have been reported to secrete higher quantities of IL17, in addition to IFN*γ*, during *Chlamydia pneumoniae* lung infections [49]. Early production of IL17 may amplify the development of Th17 response in adaptive immunity [50]. The mechanisms behind it have not been elucidated even both paracrine and/or autocrine promotion of IL17 production are suggested [51].

Several studies have focused on the role of Th17/IL17 in infectious and noninfectious diseases, while little information is available on the contribution of IL17 and Th17 to the immunopathogenesis of intracellular bacterial infections in humans. In the present review, we provided an overview of the advances of the roles and cellular mechanisms of Th17/IL17 in the host immunity against the intracellular bacterial infections (the major characteristics of pathogenic intracellular bacterial species, as well as the Th17/IL17 functions, were summarized in Table 1). A better understanding of immune complexity will contribute to the identification of disease/resistance biomarkers and influence the development of vaccines and immunotherapies for intracellular bacteria.

## 2. Th17/IL17 in Intracellular Bacterial Infection

### 2.1. *M. Tuberculosis*

It has been shown that Th17 is involved in the immune response to *M. tuberculosis* [16, 52–54]. However, the exact role of IL17 in the *Mtb* infection is still unclear. It seems like the roles of Th17 are dependent on the stage of infection, bacteria strains, or its burden. And Th17 response is dispensable for protection if predominant Th1 response is present in the primary *Mtb* infection [53, 55]. In the early developmental stage of initiating a protective immune response during *Mtb* infection, Th17 cells facilitate the recruitment of neutrophils, macrophages, and Th1 cells to the area of inflammation and participate in the control of the infection process [56]. Umemura et al. showed that Th17 is critical not only in the early activation of lung neutrophils but also in the development of Th1 responses in *Mtb* infection [15]. In addition, Khader et al. found that Th17 induced the expression of CXCl9, CXCL10, and CXCL11 chemokines, recruits IFN*γ*-producing cells, and thus ultimately restricts the reproduction of mycobacteria in macrophage in BCG vaccination model [16]. The relatively increased levels of specific cytokines such as IL6, IL21, IL1*β*, and TNF*α*, produced by mycobacteria-infected cells, may act as cofactors for Th17 differentiation [10, 57, 58].

In other studies, on the contrary, some scientists believe that Th17 response has rather pathological than protective effects because there is a connection between the progression of pulmonary *Mtb* and the hyposecretion of IL17. IL17 appeared to enhance *Mtb* dissemination from primary pulmonary infection [53]. While, at secondary disease sites, IL17 neutralized mice had less granulocyte in the lungs and resulted in less bacterial load in the spleen. Their observations suggested that IL17 impaired the host's ability to control *Mtb* infection [59, 60]. In contrast, mice infected with BCG benefited from IL17 protection [61]. One study emphasized the role of IL17 in the granuloma formation in the BCG-infected lung. They found that IL17A^−/−^ mice showed a normal level of nascent granuloma formation on day 14 but failed to develop mature granulomas on day 28 after the BCG infection in the lung. The observation implies that IL17A is required for the maturation of granuloma from the nascent to the mature stage. Furthermore, IL17A KO mice had an impaired protective response to virulent *Mtb*. So, they suggested that IL17A plays a critical role in the prevention of *Mtb* infection through the induction of mature granuloma formation [59]. In addition, IL17 was necessary for accelerated Th1 memory response and provided protection in BCG vaccinated mice [16]. IL17-produced cells responded quickly and populated the lung. That signaling was necessary for the trafficking of Th1 cells to the lungs [16]. IL17 seems important in maintaining a long lasting immune response [62]. However, recent studies showed that repeated BCG vaccination after *Mtb* infection resulted in increased IL17 production, which was responsible for the influx of granulocytes and neutrophils and lung tissue damage [63, 64]. All the data suggest that more efforts are necessary to explore the mechanisms behind the discrepancy of the role of Th17/IL17 in different *Mtb* strain infections/vaccinations.

### 2.2. *Listeria monocytogenes*

*L. monocytogenes* is a facultative intracellular bacterium that is one of most virulent foodborne pathogens. In addition, *L. monocytogenes* has been widely used as a model organism to illustrate the host immunity for intracellular bacterial infection. It has been shown that IL17A- and IL17A-producing *γδ*T cells had a beneficial effect against intracellular *L. monocytogenes* infection, not only by expansion and accumulation of innate neutrophils but also by promoting adaptive CTL responses through enhancing DC cross-presentation [44, 65, 66]. IL23 signaling controls the balance between Th1 and Th17 responses. And IL23/IL17 axis is required for an optimal immune response against *L. monocytogenes* infection [67]. IL17R^−/−^ and IL23^−/−^ mice are more susceptible to *L. monocytogenes* infection [67, 68]. In addition, administration of exogenous rIL17A [67] or adoptive transfer of IL17-producing cells [43] reduced bacterial burden in the liver of *L. monocytogenes*-infected mouse [67]. All these data support that IL17 provides a protective immunity against *L. monocytogenes*. However, in a less virulent strain infection, the host may control bacterial growth in a fashion independent of IL17A [69].

### 2.3. *Chlamydia*

*Chlamydia trachomatis* (*Ct*) is a gram-negative pathogen which causes various diseases, including cervicitis, pelvic inflammation, ectopic pregnancy, sterility, pneumoniae, and trachoma [70]. One human study showed that in *Ct* infection, both IL23 and IL17 production were dramatically increased compared to uninfected patients and both cytokines actively participate in all processes of host defense against infection [71]. An in vivo study showed that IL23-deficient mice exhibited normal susceptibility to infection and oviduct pathology. IL23 was required for the development of a *Chlamydia*-specific Th17 response in the lymph nodes and for production of IL17 in the genital tract. It is likely that IL23 plays a minimal role in the pathogenesis of *Chlamydia* infection in the mouse model [72]. Our work confirmed that enhanced IL17 production and Th17 expansion in *Ct* infection was critical for host defense against *Ct* infection. It suggested a significant detrimental impact of in vivo IL17 neutralization by anti-IL17 mAb on disease course, immune response, and dendritic cell (DC) functions. The DC from IL17-deficient mice showed lower CD40 and MHC II expression and IL12 production [18]. Our findings have been supported by the other studies [73, 74]. However, another *Chlamydia* strain, *C. pneumoniae*, has been found which impaired IL17 signaling pathway through inhibiting Act1 recruitment to the IL17R which prevented NF*κ*B activation [75].

### 2.4. *Salmonella enterica*

*Salmonella enterica* is a gram-negative bacteria species, including *Salmonella enterica* serovar Enteritidis (S. Enteritidis) and *Salmonella typhimurium*, two most abundant serotypes that cause gastroenteritis or systemic infection in human [76]. IL12/IFN*γ* and B cells contribute to host protective immunity to *Salmonella* [77, 78]. IL17 production is enhanced shortly after the *S. enterica* infection in mice model, but IL17-associated response is dispensable in the presence of an effective Th1 response. In contrast, in the absence of Th1 responses, IL23-dependent IL22 is required for protection against the sublethal doses of S. Enteritidis, but not IL17A [79, 80], while another exquisite study showed that Th17A were important for orchestrating early inflammatory responses during *S. typhimurium* colitis [19, 76].

### 2.5. *Francisella tularensis*

*F. tularensis*, a causative agent of tularemia, is ranked as a category A agent of bioterrorism by the US Center for Disease Control [81]. Inhalation is extremely dangerous and is most likely the route of bioterrorism because low doses of airborne bacteria could cause severe diseases [82, 83]. Numerous studies confirmed that IFN*γ* and Th1 cell responses are important for host control of *F. tularensis* infection and implicate IL17 in the regulation of Th1 cell immunity against *Francisella tularensis* [84, 85]. Further study showed that the IL23/Th17 pathway regulated the IL12/Th1 cell pathway and was required for protective immunity against *F. tularensis* live vaccine strain. Furthermore, the study found that IL17A induced IL12 production in dendritic cells and mediated Th1 responses. And IL17A also induced IL12 and INF*γ* production in macrophages and mediated bacterial killing [86]. So, Th17 cells play a pivotal role in immunity to *F. tularensis* infection.

## 3. Mechanisms of Th17/IL17 in the Intracellular Bacterial Infection

### 3.1. Immunomodulation of DC

**Figure 1:**
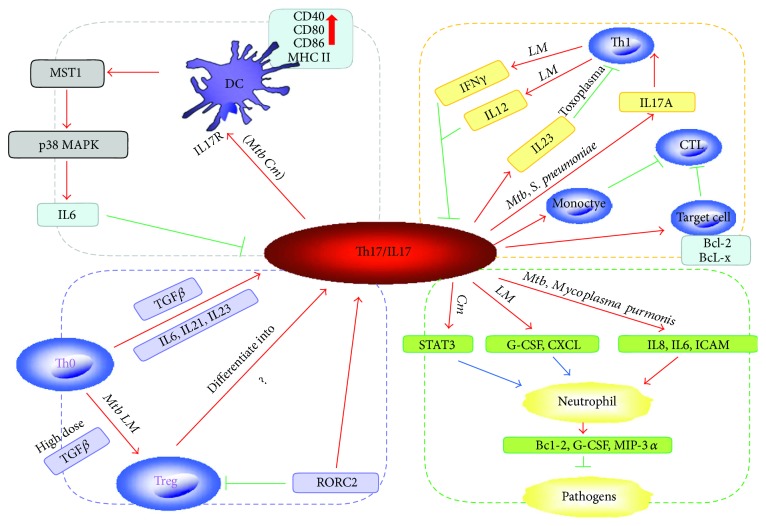
Schematic depiction of Th17/IL17 immunoregulation mechanisms.

Dendritic cells are professional APCs in priming CD4^+^ and CD8^+^ T cells [87]. After stimulation, DC could upregulate both IL17 receptor expression and the Th17 responses [50]. IL17 promoted DC differentiation through upregulating cell surface costimulatory molecule expression, such as CD40, CD80, CD86, and MHC class II, which have been reported in the organ allograft rejection [88] and airway inflammation [89, 90]. On the other hand, an exquisite study showed that mammalian sterile 20-like kinase 1 (MST1) signaling from DCs negatively regulated Th17 differentiation. MST1 deficiency in DCs increased IL17 production by CD4^+^ T cells, whereas ectopic MST1 expression in DCs inhibited it. Notably, MST1-mediated DC-dependent Th17 differentiation regulated experimental autoimmune encephalomyelitis and antifungal immunity. Mechanistically, MST1-deficient DCs promoted IL6 secretion and regulated the activation of IL6 receptor *α*/*β* and STAT3 in CD4^+^ T cells in the course of Th17 differentiation. Activation of the p38 MAPK signal was responsible for IL6 production in MST1-deficient DCs. Thus, the results defined the DC MST1-p38 MAPK signaling pathway in directing Th17 differentiation [91] (Figure 1).

Our lab investigated the effect of IL17 on the maturation of DC in the intracellular *C. muridarum* infection [18]. The DC isolated from IL17-neutralized mice showed lower CD40 and MHC II expression and IL12 production, but higher IL10 production compared with those from sham-treated mice. In in vitro DC-T cell coculture systems, DC isolated from IL17-neutralized mice showed less maturation and induced higher IL4, but lower IFN*γ* production by Ag-specific T cells than by those from sham-treated mice in cell priming and reaction settings. Adoptive transfer of DC isolated from IL17-neutralized mice, unlike those from sham-treated mice, failed to protect the recipients against challenge infection. These findings provided evidence that IL17/Th17 can modulate DC function and thus provide protection against *Cm*.

*Mtb* vaccine study based on liposome demonstrated that Ag85B-ESAT-6/CAF01 can induce protective Th17 and Th1 immune response through prolonged DC uptake and activation [92]. Ag85B-ESAT-6/CAF01 vaccination dramatically decreased the post challenge bacterial growth of BCG and induced strong Th1 and Th17 responses in neonatal and adult groups. DC in the draining LN associated with protection were more mature regarding the high expression of CD40 and CD86, and the activated DC were recovered several days after immunization [92]. However, they did not directly examine the relationship of IL17 and maturation of DC in their model. Another study directly showed that *Mtb* induced monocyte-derived human DC maturation through increasing the expression of CD80, CD86, CD40, CD83, HLA-DR, and HLA-I, as well as cytokine production including IL23. Dectin-1 molecule engaged on DC promotes Th17 response, where DC-SIGN and MR costimulation limited Th17 generation and favored Th1 responses [93, 94].

In virulent *L. monocytogenes* infection, Xu et al. found that IL17A could upregulate the MHC class I molecular H2-K^b^ expressed on DC while no effects on CD40, CD80, CD86, and MHCII. They analyzed the DC phenotype and function in IL17A-deficient mice after *LM* infection. The data showed that the absolute number of DC, especially CD8^+^ DC as the major DC subset contributing to the cross-presentation [95], was not changed. However, DC deficiency in IL17A signaling lost the potential to promote OT-I T cell activation and proliferation [44]. They implied in their study that IL17A, instead of IL17F, can enhance cross-presentation of DC in vivo and in vitro [44].

Cytokine production is an important characteristic of DC. Lin et al. compared cytokine production in *F. tularensis*-infected DC. They found that IL17A-treated *F. tularensis* LVS-stimulated BMDCs resulted in the significantly enhanced IL12 production in comparison to *F. tularensis* LVS treatment alone. However, IL17F and IL22 treatment did not impact IL12 production by the BMDC [86]. Using IL17A neutralized antibody experiment also supported that IL17A was important to promote IL12 produced by DC in *Cm* infection [18, 96].

### 3.2. Immunoregulatory Function of Th17

Th17 lymphocytes perform an immunoregulatory function, which produce a unique range of cytokines and chemokines as mentioned before. Besides, Th17 also induces the production of proinflammatory cytokines (TNF*α*, IL1*β*, G-CSF, and IL6) by macrophage and expression of CC and CXC chemokine receptors. Therefore, Th17 contributes to the recruitment and expansion of cells of innate immunity. Of all cytokines, IL17 more strongly stimulates the production of human Bd2, G-CSF, and MIP-3*α*, known to be the main humoral components of the innate immunity of the respiratory tract. It provides effective protection against pathogens. The Bd2 and MIP-3a highly expressed on neutrophil, which was migrating from peripheral blood into the tissue and backwards, were activated in response to IL17A [97, 98] (Figure 1).

#### 3.2.1. Recruitment of Neutrophil

The crosstalk between Th17 and neutrophils has been investigated in many diseases. The fact that transfer of IL17 cDNA to the mice liver or intraperitoneal injection of IL17 in mice increased neutrophil recruitment [99] suggested that IL17 contributed to the local neutrophil accumulation [100]. The mechanisms behind the IL17-mediated recruitment of neutrophil are not fully understood, but at least three mechanisms have been reported. Firstly, indirect chemoattraction is involved. Th17 cells or *γδ* T cells derived IL17 mobilize neutrophils via induction of chemokine/cytokines secretion by epithelial and endothelial cells. IL17 regulates granulocyte colony-stimulating factor (G-CSF) produced by the epithelial cells and thus promotes expansion of neutrophil. Secondly, IL17 can upregulate epithelial cells expressing chemokines, like CXCL1, CXCL2, and CXCL8 [101, 102]. The biologically active CXCL8 is a strong chemoattractant for neutrophils [103]. In addition, a number of cytokines released by the Th17, such as IL8, IL6, and adhesion molecule ICAM-1, are related to the maturation and activation of neutrophils [104, 105]. And thirdly, some studies showed that IL17 inducing STAT3 activation is a necessary step in neutrophil recruitment, and STAT3 acts as a link between IL17-mediated endothelial cell activation and neutrophil recruitment [106] (Figure 1).

The direct relationship of Th17 and neutrophil recruitment in *Mtb* infection has been rarely reported. And the fact showed that both IL17 production and neutrophil recruitment have been found in *Mtb* infection. The role of neutrophil in tuberculosis infection is controversial. Neutrophil might help limit bacterial spread, but intense neutrophilia is an important factor contributing to inflammatory immunopathology [16, 53, 107]. Further study showed that during chronic infection with *Mtb*, neutrophils were recruited to the lung in two waves after intranasal infection with virulent *Mtb* or the live attenuated vaccine strain BCG. A first wave of neutrophils was swiftly recruited, followed by a subsequent adaptive wave that reached the lung together with IFN*γ*- and IL17A-producing T cells. Interestingly, the adaptive wave was critically dependent on the expression of IL17RA, the receptor for IL17A expressed in nonhematopoietic cells [108].

It is well known that neutrophils are required for elimination of the *L. monocytogenes* and for survival of the host. Neutrophil-deficient mice have increased bacterial loads in their spleen and liver [109, 110]. Sieve et al. demonstrated that IL17A induced in the *Mycoplasma pulmonis* infection provided a cross protection against subsequent *L. monocytogenes* infection. And this IL17A-mediated protection was mediated through increased recruitment of neutrophils [111]. IL17 facilitated recruitment of neutrophils to the infective sites due to enhanced multitude of cytokine and chemokine production, like G-CSF, GM-CSF [112], CXCL1, and CXCR2 [113]. Collectively, these data suggested that IL17 was required for optimal neutrophil recruitment and host resistance in *LM* infection. Lin also found that similar mechanisms of IL17A recruited neutrophil to the lung through induction of G-CSF in *F. tularensis* pulmonary infection model [67, 86] (Figure 1).

Our study of the interaction of Th17/IL17 on the recruitment of neutrophils in *C. Muridarum* (*Cm*) infected mice showed that IL17 neutralization induced less neutrophil inflammation but suffered more severe infections. Exogenous IL17 treatment significantly enhanced the neutrophil infiltration in the lung in response to *Cm* infection [73]. Neutrophil alone may not be efficient in controlling *Cm* pulmonary infection [114]. The exact role of neutrophil in the control of *Cm* infection is not clearly understood.

The contribution of IL17 on the neutrophil recruitment is also reported in the *S. enteria* infection. *S. enteria*-infected WT mice have a relatively higher portion of CD11b^+^Gr1^+^ cells than IL17A^−/−^ mice, while the portion of CD4 T cells, CD8 T cells, macrophages, and dendritic cells have no changes [19]. Neutrophils contributed to the host resistance to S. Enteritidis, too [76, 99, 115].

#### 3.2.2. Promoting CTL Response

Cytotoxic T cells (also known as cytotoxic T lymphocytes (CTLs)), one of T cell subsets, release perforin and/or granulysin which causes the infected cells to burst or lyse. The role of IL17 in CTL function is not fully clear. Some studies showed that IL17 could activate monocytes to express B7-H1. Consequently, the B7-H1^+^ monocyte cell effectively suppressed cytotoxic T cell immunity in vitro. It suggested that IL17 selectively impaired the generation and functions of CLT [116]. A similar study found that IL17 promoted the expression of Bcl-2 and Bcl-x, and thus prevented cellular apoptosis at a much lower concentration and inhibited cytotoxic T cell function [117] (Figure 1).

### 3.3. Crosstalk of Th1 and Th17

As professional antigen presentation cells, DCs rapidly produce both IL12 and IL23 when stimulated with antigens [53]. IL12 is known for the ability to promote Th1, while IL23 is necessary for Th17 differentiation. IL12 is comprised of IL12p40 and IL12p35, while IL23 shares an IL12p40 subunit with IL12. IL12p40 is covalently bound to p19 subunit that is implicated in the induction of Th17. The cross regulation of these two cytokines is critical for the balance of Th1 and Th17 [118] (Figure 1).

#### 3.3.1. IL17 Promotes Th1 Responses

IL17 induces a protective Th1 response against intracellular pathogens [16, 18, 22, 86]. Th1 cells are essential for the host to control mycobacterial replication by activating macrophage and CD8^+^ cytotoxic cells [119–121]. But how Th17 influences Th1 in *Mtb* infection is still an interesting question [16]. The dynamics of Th1 and Th17 in *Mtb* infection is different. IL17 is produced very early in *Mtb* infection and BCG vaccination that IL17 recall preceded Th1 responses. And Th17 populated the lung and triggered the chemokine production that recruited IFN-*γ*^+^ Th1 cell, which ultimately limited bacterial growth [16]. IL17 supplement can restore the Th1 recall response in IL23-deficient mice while IL17 depletion reduced the Th1 responses.

In the absence of IL12p70, IL23 is essential for the generation of Th1 cells. There are reports showing that IL23 can compensate for the absence of IL12p70 in both *Tuberculosis* [53] and *Toxoplasma* infection [122], that exogenous IL23 was able to control pathogen burden in IL12p40-deficient mice through promoting Th1 response. Both models suggested that compensatory IL23 response may protect the host from infection. The same was in *Cm*-infected mice model [18]. Our study showed that IL17-neutralized mice exhibited reduced Th1 antigen-specific immune responses and lower Th1-promoting cytokine production in both spleen and dLN. A significant contributing role of IL17/Th17 in enhancing type 1 cytokine responses in both CD4 and CD8T cells during *Cm* infection is supported. Another intracellular infection model also confirmed that IL23-Th17 pathway regulated the IL12-Th1 cell pathway [86]. They found that Th17/IL17 was required for protective immunity against *F. tularensis* live vaccine strain. Further mechanism investigation showed that IL17A, but not IL17F or IL22, induced IL12 production in both dendritic cells and macrophages and mediated Th1 responses. Exogenous delivery of IL17A can rescue the IFN*γ* levels in *F. tularensis*-infected lungs [86].

Th1 and Th17 pathways are compensatory to each other in some intracellular bacterial infection. Meeks et al. proposed that both Th1 and Th17 responses are activated in the *LM* infection even if they did not directly compare the two pathways. They demonstrated that activated IL12/IFN*γ* axis was essential for the macrophage activation and IL23/IL17 axis influenced the neutrophil recruitment to the infection site. They suggested the IL17 functions as a complementary, but separate, branch of the immune system to the IL12 during *LM* infection [67]. Failure of either of them can increase host susceptibility to *LM* infection [67]. Schulz et al. also suggested that Th17 complements the Th1 response which is essential for protective immunity in both human and mice in their study of the *S. enterica* infection. The IL17A-deficient mice showed reduced and delayed clearance of bacteria but had no impact on the Th1 responses [19]. A further study suggested that immunization of mice with *S. pneumoniae* induced protective immunity that depended on IL17A and CD4^+^ T cells. However, this immunity may be short-lived since IL17A-producing CD4^+^ effector T cells did not survive to become memory cells [123] (Figure 1).

#### 3.3.2. Th1/Th17 Cross Regulate Each Other

Other studies also suggested that Th17 cells can negatively regulate Th1 in some infections. In BCG-infected mice, IL17 limits IL12 production while it enhances IL23 production. IFN*γ* can increases IL12p70 but reduce IL23 [124]. Cells with the ability to secrete IFN*γ* and IL17 concomitantly (T_H_17/T_H_1 cells) have been described in inflammatory diseases in mice [125] and in humans [126]. And Yeh et al. demonstrate that IFN*γ* inhibits Th17 differentiation and functions in a STAT1-dependent and Tbet-independent manner [118]. Moreover, the transition from IL17-positive cells to IFN*γ*-positive cells in the presence of IL12 has also been reported [127, 128]. Double-positive cells have been found recently in *Cm*-infected model [129]. Together, all these findings illustrate a biological function for IL17A in regulating Th1 cell immunity and host responses to intracellular pathogens (Figure 1).

### 3.4. Interaction with Regulatory T Cells (Treg)

The participation of TGF-*β* in the differentiation of Th17 cells makes Th17 lineage development closely related to the regulatory T cells since TGF-*β* also promotes the FoxP3^+^ Treg differentiation. The balance between these subpopulations of lymphocytes is established due to the antagonistic interaction of ROR*γ*t (encoded by RORC2 in humans) and FoxP3 transcription factors [130, 131]. However, the antagonistic relationship does not exclude their simultaneous expression in the CD4 T cells as demonstrated that the transcript factors for Th17 (ROR*γ*t) or Treg (FoxP3) were found coexpressed in naive CD4^+^ T cells [130]. Reciprocal regulations of Th17 and Treg cells are controlled by the presence of specific cytokines in the differential environment [130, 132]. TGF-*β* is required for the expression of both FoxP3 and ROR*γ*t. A high dose of TGF-*β* can orchestrate Treg cell differentiation through preferably inducing FoxP3 expression in T cells and thus endow their cells with regulatory/suppressor capacity [133]. IL6 also plays a pivotal role to control FoxP3/ROR*γ*t balance, thus directing the balance between the generation of Tregs and Th17. The synergization of IL6 or IL21 and IL23 with TGF-*β* can relieve FoxP3-mediated inhibition of ROR*γ*t [130] and promote Th17 differentiation in both in vivo and in vitro studies [24, 132, 134–137]. It was reported that RORC2 inhibited the FoxP3 expression due to the competition with the NFAT transcription factor, which is essential for the FoxP3 gene activation [138]. Koenen et al. showed that human Treg cells were capable of differentiating into IL17-producing T cells [139]. It implied that human Treg cells not only act as a suppressor but also has additional proinflammatory functions, even mechanisms that are currently unknown. And IRAK is an important intracellular kinase to direct the differentiation of naïve CD4^+^ T cells into Th17 or Treg [140] (Figure 1).

The interdependent regulation of Treg and Th17 cells is well characterized in some human inflammatory diseases [141, 142]. Treg may restrict the overwhelming immune response to protect the host from tissue damage caused by the effector cells. But during *Mtb* infection, Treg may be deleterious since they downregulate DC antigen presentation and macrophage activity and therefore release the *Mtb* replication control [143–145]. Th17 is activated during early *Mtb* infection. But the interaction of Treg and Th17 is inconsistent. In latent *Mtb* infection (defined by TST positivity), there is a study showing a clear inverse relationship of Treg and Th17. They found that depletion of CD4^+^CD25^+^ T cells dramatically reversed *Mtb*-specific Th17 inhibition in TST-positive but not TST-negative patients. They suggested that downregulation of Th17 responses by Treg cells played a vital role in mediating resistance to latent infection [146]. However, recent human study showed that Treg only suppressed the activated Th1 immune responses, while it had no effect in the inhibition of the proinflammatory Th17 responses in activated and latent *Mtb* infection in in vitro PBMC culture [147]. A similar conclusion has been reported in *LM* infection. IL17A was excluded to interfere with Treg cells [44].

It should be noted that data on the regulation of the differentiation of Th17 and Treg now were obtained from animal models. In the case of infectious diseases in humans, the regulatory mechanisms of these process, in general, remain unclear or are not studied at all.

## 4. Conclusion

The new discoveries of the functions of Th17/IL17 in host immunity have been accumulated rapidly in the last few years. Despite the importance and functional significance of Th17 lymphocytes, their clinical significance and regulatory mechanisms in the development of anti-intracellular bacterial immunity are still not well understood. An analysis of the role of Th17 cells in the immunopathogenesis in intracellular bacterial infections is of indisputable scientific interest. Subsequently, understanding the Th17/IL17 responses and its interaction and regulation with other immune repertoire provides critical insights into the host immune defense in infectious diseases. It would facilitate the development of new effective immunomodulatory strategies for the treatment and prophylactic in bacterial infection.

## Figures and Tables

**Table 1:** The major characteristics of pathogenic intracellular bacterial species.

Serial number	Bacterial species	Gram staining	Facultative/obligatory	Diseases	Function of Th17/IL17
1	*M. tuberculosis*	Positive	Obligatory	Pulmonary infection, pleurisy, tuberculous pericarditis, bone tuberculosis, tubercular meningitis, tuberculous arthritis	(i) Recruit neutrophils, macrophages, Th1 cells and IFN*γ*-producing cells (ii) Accelerate Th1 memory response

2	*Listeria monocytogenes*	Positive	Facultative	Septicemia, meningitis, monocytosis	(i) Promote adaptive CTL responses (ii) Enhance DC cross-presentation (iii) Accumulate innate neutrophils

3	(i) *Chlamydia trachomatis* (ii) *Chlamydia pneumoniae*	Negative	Obligatory	Pelvic inflammatory disease, trachoma, pneumoniae	Promote DC functions

4	(i) *Salmonella enterica* serovar Enteritidis (ii) *Salmonella typhimurium*	Negative	Facultative	Typhoid fever, paratyphoid fever, Enteritidis	IL23 is required for protection against the sublethal doses of S. Enteritidis

5	*Francisella tularensis*	Negative	Facultative	Tularemia	(i) Regulate the IL12-Th1 cell pathway (ii) Induce IL12 and INF-*γ* production
